# Drug Resistance in the Microaerophilic Parasite *Giardia lamblia*

**DOI:** 10.1007/s40475-015-0051-1

**Published:** 2015-07-07

**Authors:** David Leitsch

**Affiliations:** Institute of Parasitology, Vetsuisse Faculty, University of Berne, Länggass-Strasse 122, CH-3012 Berne, Switzerland

**Keywords:** *Giardia lamblia*, Dysentery, Chemotherapy, Drug resistance

## Abstract

The microaerophilic parasite *Giardia lamblia* is a causative agent of dysentery affecting hundreds of millions of people around the globe every year. The symptoms of the disease, commonly referred to as giardiasis, are diarrhea, nausea, and malabsorption. Treatment of giardiasis is exclusively based on chemotherapy with antigiardial drugs, including metronidazole, albendazole, and nitazoxanide. In this review, all drugs currently used in the treatment of *Giardia* infections are discussed with a special emphasis on treatment failure and drug resistance.

## Introduction

*Giardia lamblia* (syn. *intestinalis*, *duodenalis*) is a worldwide occurring protist parasite that causes a form of dysentery commonly referred to as giardiasis [1]. Although *Giardia* infections frequently present without symptoms and are often self-limiting, severe gastrointestinal symptoms such as diarrhea, nausea, bloating, or malabsorption can also ensue. Post-infection symptoms such as lactose intolerance [2] or irritable bowel syndrome are not rare [3]. With as much as 33 % of the population in developing countries having a record of giardiasis [4], *G. lamblia* is considered to be the most common protist parasite of the human gastrointestinal tract. It preferably colonizes the small intestine and has a two-staged life cycle. The acid-resistant, metabolically widely inactive cyst is ingested by the host and passes through the stomach into the small intestine. There it excysts and develops into the trophozoite which is the actively feeding and pathogenic stage. The trophozoite either moves freely in the lumen or attaches to the intestinal mucosa. A given proportion of the trophozoite population undergoes development into cysts which are excreted in the feces and contaminate water or food. Upon ingestion of cysts by another host, a new cycle of infection can commence.

**Fig. 1:**
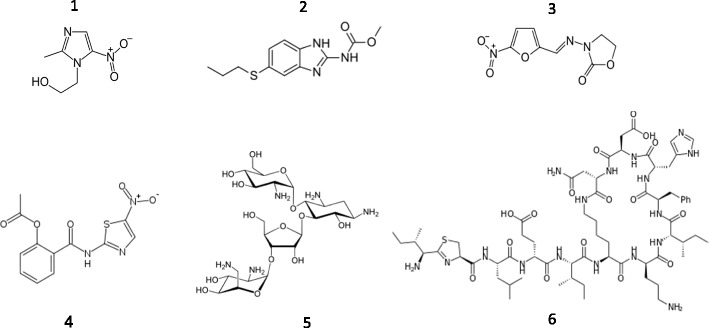
Antigiardial drugs: metronidazole (1), albendazole (2), furazolidone (3), nitazoxanide (4), paromomycin (5), and bacitracin (6). All images are taken from wikipedia.org

As no prophylaxis against *Giardia* infections is available, countermeasures are limited to chemotherapy of established infections. Fortunately, a comparably large selection of drugs is available for the treatment of giardiasis. The most commonly prescribed drugs are the 5-nitroimidazoles metronidazole and tinidazole and the benzimidazole albendazole (Fig. 1). Other drugs used are quinacrine, nitazoxanide, furazolidone, mebendazole, paromomycin, and bacitracin zinc (Fig. 1). Auranofin, an approved antirheumatic [5•], and the orphan drug fumagillin [6•] are highly effective against *G. lamblia* and might be introduced as antigiardial drugs in the near future.

In most of the cases (about 90 %), treatment regimens with either metronidazole or albendazole alone are effective, but recalcitrant giardiasis, sometimes due to drug resistance, is not rare [7]. In such cases, combination therapy regimens of metronidazole and albendazole or quinacrine are highly effective [8, 9]. However, it cannot be denied that drug resistance does occur in *G. lamblia*, which is a matter of concern due to the frequent occurrence of this parasite.

This review will give an overview over the most common drugs in use against *G. lamblia*, occurrence and mechanisms of drug resistance, and other circumstances that may lead to the failure of treatment.

## Antigiardial Drugs

The arsenal of antigiardial drugs is fortunately rather large although only few drugs are used in daily practice. In fact, in some countries, e.g., Norway [10], only metronidazole is approved for the treatment of giardiasis. In addition to the above-mentioned compounds (Fig. 1), pentamidines [11], chloroquine, DL-propanolol, propolis, and ozonized sunflower oil have been successfully tested against *G. lamblia* in vitro and in vivo [12].

### Metronidazole, Tinidazole, and Ornidazole

Metronidazole (Fig. 1) is a 5-nitroimidazole drug which was specifically developed in the late 1950s against another microaerophilic parasite, *Trichomonas vaginalis* [13]. Soon after its discovery, metronidazole was also successfully tested against numerous other anaerobic and microaerophilic pathogens, including *G. lamblia* [14], and is nowadays listed among the “essential medicines” by the WHO. Metronidazole is, in fact, a prodrug which needs to be reduced at its nitro group in order to become toxic [15]. Due to the very low redox potential of metronidazole [16], this only quantitatively occurs in microaerophilic and anaerobic organisms which have a strongly reductive physiology [17]. In the 13th report on carcinogens issued by the NIH [18], metronidazole has been classified as carcinogenic because it causes tumors in mice when administered in very high doses. However, no link between metronidazole treatment and cancer has so far been observed in humans despite its frequent use. Thus, although inconvenient side effects are regularly reported, metronidazole is a rather safe drug in humans and animals. Treatment is carried out for 5–10 days with one or two doses per day [12].

Metronidazole damages DNA in microaerophiles and anaerobes [19], obviously by introducing double strand breaks [20•]. In addition, metronidazole binds to free thiols, such as cysteine, which has a similar role in many microaerophilic parasites as glutathione has in mammalian cells [21–24]. Metronidazole also forms covalent adducts with cysteines in proteins. Specifically, metronidazole targets the redox enzyme thioredoxin reductase in *Entamoeba histolytica*, *Trichomonas vaginalis*, and *G. lamblia* and inhibits the enzyme’s function as a disulfide reductase [21, 22, 24]. All these events result in severe oxidative stress. This is further corroborated by the observation that cysteine, added to the growth medium, protects *Giardia* from metronidazole [25]. In *G. lamblia*, metronidazole also leads to the degradation of elongation factor 1-γ (EF1-γ), an enzyme involved in protein translation [23]. The mode of action of other 5-nitroimidazoles, e.g., tinidazole and ornidazole, is very likely to be identical [21]. However, tinidazole and ornidazole are more efficacious than metronidazole because they have considerably longer serum half-lives than metronidazole [12] and can be administered as single doses of 1 or 2 g. As a result, they have a better patient compliance than metronidazole.

### Albendazole and Mebendazole

The benzimidazole drug albendazole (Fig. 1) was approved in 1983 for the treatment of helminth infections and was soon found to be efficacious against *G. lamblia* as well [26–28]. When given in single 400 mg doses for 5 days, it is equally effective as metronidazole but has fewer side effects because it is poorly absorbed in the human gut [29]. Consequently, albendazole therapy against giardiasis is an alternative to metronidazole therapy. Another benzimidazole, mebendazole, was also found to be effective against *G. lamblia* [27]. Treatment courses with mebendazole, however, have been reported to be variably effective [30–33]. For both, albendazole and mebendazole, teratogenic effects in rats were reported [12].

One confirmed target of benzimidazoles is β-tubulin [27, 3], a major component of the cytoskeleton in *G. lamblia* [35]. Albendazole and mebendazole bind very efficiently to β-tubulin in susceptible organisms [36], including *G. lamblia*, thereby leading to the inhibition of cytoskeleton polymerization and, consequently, to severe structural defects [37].

### Nitazoxanide

The nitrothiazolide compound nitazoxanide (Fig. 1) was first described as antihelmintic [38] but later also found to be active against a range of other parasites including *Giardia lamblia* [39]. In 2004, it was approved in the USA for the pediatric treatment of giardiasis. As is the case with 5-nitroimidazoles, nitazoxanide has to be reduced at the nitro group for activity in *Giardia* [40]. Nitazoxanide is usually prescribed for 3 days and well tolerated because it is poorly absorbed in the intestine [12]. No evidence for any mutagenic or teratogenic activity of nitazoxanide has been found so far.

The mode of action of nitazoxanide is very likely to be multifactorial. Nitazoxanide strongly inhibits the pyruvate decarboxylating enzyme pyruvate:ferredoxin oxidoreductase (PFOR), an enzyme of central physiological importance in *G. lamblia* [41]. However, nitazoxanide also inhibits other enzymes, such as nitroreductase 1 [42], a presumptive quinone reductase [43], and compromises the integrity of the cell by causing lesions in the ventral cell membrane and inducing vacuolization [40].

### Quinacrine

The acridine drug quinacrine (Fig. 1) was originally introduced as an antimalarial but also used for the treatment of giardiasis after the Second World War [34]. The drug is very effective but must be given over 5–10 days and frequently causes side effects such as nausea or discoloration of the skin. Its mode of action has been scarcely studied, but it was found to inhibit nucleic acid synthesis [44] In *G. lamblia*, however, no evidence was found that quinacrine accumulates in the nucleus [45]. As is the case with metronidazole, quinacrine toxicity is counteracted by cysteine [25], suggesting that the drug’s mode of action includes induction of oxidative stress. Currently, quinacrine is only used as a second-line therapeutic in combination with metronidazole or albendazole.

### Paromomycin

Effectiveness of the aminoglycoside paromomycin (Fig. 1) against *G. lamblia* was first reported in 1989 [46]. The efficacy of the drug, however, greatly varies among laboratories [46, 47], and in one instance, no effect of paromomycin on *G. lamblia* was observed at all [48]. Paromomycin has been rarely used in the treatment of giardiasis but its particular advantage is that it is practically not absorbed by the intestine, rendering it safe for use during pregnancy [34]. It is effective as a second-line drug in combination with metronidazole [49].

### Furazolidone

Like the 5-nitroimidazoles and nitazoxanide, the nitrofuran furazolidone (Fig. 1) is a prodrug whose nitro group must be reduced in order to be rendered active [50, 51]. It has a higher redox potential than 5-nitroimidazoles and is therefore more efficiently reduced in microaerophilic parasites [22, 52]. The reduced intermediates of furazolidone likely damage cell constituents such as DNA and proteins [34]. A related nitrofuran, nifurtimox, was reported to bind to thiol groups [53] and to lower free thiol concentrations in *Trypanosoma cruzi* [54].

Furazolidone is clinically highly effective when administered for 7–10 days, but shorter courses yield lower cure rates. One great advantage of furazolidone is its availability as a liquid suspension which has a higher compliance with children.

### Bacitracin Zinc

The polypeptide antibiotic bacitracin (Fig. 1) was found to be effective against *G. lamblia* in 1994 [55] and was even several-fold more so in combination with zinc. To date, only one clinical trial for treatment of giardiasis with bacitracin zinc was performed [56]. Although bacitracin zinc treatment was effective, it had to be continued for 10 days which negatively affects patient compliance [34].

## Treatment Failure and Drug Resistance in *G. lamblia*

Most clinical studies on chemotherapy of giardiasis demonstrate that, regardless of the drug used, cure rates are normally below 100 %. Apart from drug resistance, other factors can also be responsible for treatment failure, such as reinfection, insufficient amounts of drug administered, immunosuppression, or sequestration in the gallbladder or the pancreas [9]. If two or more courses of treatment with a given drug are unsuccessful, it is recommended to either use another drug alone or in combination with the first drug [9, 34]. Most of the refractory cases can be ultimately cured with the arsenal of antigiardial drugs available. This is very well illustrated in a careful study on a chosen treatment regime during an outbreak of giardiasis in Norway. A treatment ladder was applied in order to minimize the number of patients remaining uncured after treatment [8]. Thirty-eight individuals out of more than 1200 treated in total were not cured from giardiasis after one to three courses of metronidazole therapy. As a next step, the refractory cases were treated with a combination of metronidazole and albendazole, resulting in the cure of 30 patients. The eight patients, neither responding to metronidazole nor to albendazole, were treated with paromomycin, resulting in the cure of four. The remaining four patients were finally cured after 3 weeks of treatment with a combination of quinacrine and high doses of metronidazole. Importantly, the combination of metronidazole and quinacrine was also found by others to be effective in eliminating refractory *G. lamblia* infections [9, 57].

It is important to note that *G. lamblia* isolated from patients with refractory giardiasis do not always display drug resistance in vitro [57], suggesting some physiological influence of the human host on the success of a given chemotherapy. It is, therefore, important to rule out that people with recurrent symptoms have not been re-infected and that they are not suffering from lactose intolerance or irritable bowel syndrome which frequently evolves after giardiasis [2, 3].

No reports or studies on resistance to the more rarely used antigiardial drugs, bacitracin zinc and paromomycin, have been published, but resistance in *G. lamblia* to 5-nitroimidazoles, albendazole, nitazoxanide, quinacrine, and furazolidone has been studied to varying extent.

### Resistance to Metronidazole and Other 5-nitroimidazoles

Metronidazole resistance has been intensely studied in microaerophilic and anaerobic pathogens, including *G. lamblia*. Indeed, resistant *Giardia* strains have been repeatedly isolated from patients with refractory giardiasis [58, 59]. In addition, resistance to metronidazole and other 5-nitroimidazoles is relatively easy to induce in the laboratory by exposing *Giardia* to incremental doses of drug or to UV light [7, 42, 60–62]. Eventually, tolerance to metronidazole can be increased to more than the 100-fold the IC_50_ of the drug in sensitive isolates [61].

The mechanisms of metronidazole resistance have been widely discussed throughout the last 40 years, but the observations made are often conflicting and difficult to put into perspective. Two factors that have been understood as being central for the reduction, i.e., activation of 5-nitroimidazoles, are pyruvate ferredoxin oxidoreductase (PFOR) and its cofactor ferredoxin which are almost always present in microaerophilic and anaerobic organisms [63]. As shown in several organisms, including *Giardia*, ferredoxin is reduced by PFOR and can transfer an electron to the nitro group of metronidazole [16, 64–66]. In accordance, slightly reduced PFOR activities were measured in *Giardia* stocks with reduced sensitivity to metronidazole [67] and some metronidazole-resistant strains generated in the laboratory, e.g., BRIS/83/HEPU106-2ID_10_, have only remnant PFOR activity [68, 69]. Further, a knock-down of PFOR expression using hammerhead ribozymes rendered *Giardia* resistant to metronidazole [70]. In contrast, however, metronidazole-resistant cell lines with an intact PFOR/ferredoxin pathway, e.g., BRIS/87/HEPU/713-M3, were described [69]. Moreover, *Giardia* cell lines resistant to C17, a second-generation 5-nitroimidazole [71], are highly cross-resistant to metronidazole but have fully functional PFOR and ferredoxin [69]. Thus, inactivation of the PFOR/ferredoxin pathway is not a prerequisite for 5-nitromidazole resistance. In a series of research papers, it was shown that nitroreductase 1 and 2 could have an important role in the metabolism of metronidazole in *Giardia*. Interestingly, nitroreductase 1 can activate metronidazole to reactive metabolites [72], whereas nitroreductase 2 has, conversely, a protective effect, possibly through fully reducing the drug’s nitro group with six electrons to a non-toxic amino group [73•]. In accordance with this notion, expression of nitroreductase 1 was found to be downregulated in metronidazole-resistant laboratory cell lines [72, 73•], whereas nitroreductase 2 was found to be upregulated [73•]. Furthermore, overexpression of nitroreductase 2 renders *Giardia* more tolerant to metronidazole [73•]. Expression of thioredoxin reductase (TrxR) [21, 22, 69], however, i.e., another factor that can reduce 5-nitroimidazoles, was not found to be affected in metronidazole-resistant *Giardia* cell lines [69].

In any case, it is certain that reduced metronidazole sensitivity in clinical *Giardia* isolates on the one hand and fully developed metronidazole resistance induced in the laboratory on the other are caused by different underlying mechanisms. Metronidazole-resistant cell lines generated in the lab do hardly establish infections in mice, indicating that the physiological adaptations necessary to tolerate high doses of metronidazole have high fitness costs [74]. Also in another microaerophilic parasite, *Trichomonas vaginalis*, metronidazole resistance in clinical isolates is fundamentally different from metronidazole resistance generated in the laboratory [75–78]. In metronidazole-resistant clinical *T. vaginali*s isolates, no downregulation or inactivation of factors that reduce 5-nitroimidazoles can be observed [23, 76, 79]. Instead, the oxygen scavenging capability is impaired [76], leading to higher intracellular oxygen concentrations. Since oxygen quickly re-oxidizes nitroradical anions to the non-toxic prodrug, these strains are highly resistant to metronidazole if oxygen is present [80] but normally susceptible to the drug when oxygen levels are low [75]. Presumably, downregulation of flavin reductase 1 [81], which reduces oxygen to hydrogen peroxide by using FMN and NADPH as cofactors, is a key event in the development of clinical metronidazole resistance in *T. vaginalis*. NADPH-dependent reduction of oxygen via FMN can also be observed in *G. lamblia* [68] but is catalyzed by another, yet undescribed, enzyme because *T. vaginalis* flavin reductase 1 has no homologue in the *G. lamblia* genome. Confusingly, this enzyme activity is downregulated in metronidazole-resistant *G. lamblia* laboratory cell lines [69] but strongly upregulated in clinical isolates with reduced metronidazole sensitivity [68]. Thus, for all the research efforts of the last decades, our understanding of metronidazole resistance in *G. lamblia* is still incomplete at best.

### Resistance to Nitazoxanide

Although nitazoxanide resistance has not been observed yet in the clinic, it can be induced in the laboratory [42]. In one study, a nitazoxanide-resistant cell line was strongly cross-resistant to metronidazole, whereas a cell line with induced metronidazole resistance was fully susceptible to nitazoxanide [42], indicating that cross resistance between both drugs is not necessarily reciprocal. In another study, however, laboratory cell lines, adapted to high concentrations of metronidazole, also displayed a similar level of resistance towards nitazoxanide [74]. An expression study on the nitazoxanide-resistant cell line showed several protein chaperones such as Hsp70, Hsp90, and Cpn60, and several surface antigens to be upregulated in expression [82]. Levels of PFOR were practically unaltered in nitazoxanide-resistant cell lines [42], but nitroreductase 1 was found downregulated [72]. Overexpression of nitroreductase 2, however, has no protective effect *in Giardia* against nitazoxanide [73•]. In summary, resistance mechanisms against metronidazole and nitazoxanide do partially overlap but are not identical.

### Resistance to Albendazole

Resistance to albendazole has been repeatedly observed in clinical isolates [58, 59] and in the laboratory [62, 83–85]. Indeed, *Giardia* can be adapted to concentrations of albendazole that are about 1000-fold higher than the IC_50_ of the drug in susceptible stocks [85]. In helminths, benzimidazole resistance is usually conferred by mutations in the β-tubulin gene, preferably at amino acid positions 167 and 200 [86]. In *Giardia*, however, conflicting observations have been made in this regard. In one study, an altered amino acid sequence of β-tubulin upon extended exposure to increasing concentrations of albendazole was identified [84], whereas others found no alterations in the sequence [45, 62]. Instead, chromosomal rearrangements, cytoskeletal aberrations [45], and the altered expression of several genes, including a VSP-like protein [62], constituents of the ventral disk (α2-giardin) or various enzymes such as NADH oxidase and triosephosphate isomerase [85], were found in albendazole-resistant cell lines. Interestingly, also PFOR was reported to be upregulated in albendazole-resistant cell lines [62]. The implications of these findings are still unclear as yet, but they suggest that benzimidazoles probably target a more diverse group of molecules in *G. lamblia* than in helminths.

### Resistance to Furazolidone

Based on electron paramagnetic resonance (EPR) spectra, the enzyme NADH oxidase was suggested to be an activator of furazolidone in *G. lamblia* by donating an electron to the drug’s nitro group [52]. Reduction of the nitro group gives rise to a nitroradical anion which is believed to be the actual toxic agent. However, although resistance to furazolidone can be induced in vitro [61], no correlation between NADH oxidase activity and furazolidone resistance has been reported as yet. Also, PFOR activity was not altered in furazolidone-resistant *Giardia* [15]. In fact, due to the relatively high reduction potential of nitrofurans, at least in comparison with nitroimidazoles, quantitative reduction of furazolidone might already take place as soon as reduced flavin (either free or bound to proteins as cofactors) is present in the cell [87].

In clinical isolates which demonstrate reduced sensitivity to furazolidone, thiol-dependent peroxidase and reductase activities were reported to be increased [67]. These activities can be attributed most likely to peroxiredoxin and thioredoxin reductase, respectively. It is reasonable to assume that higher levels of these enzymes render *Giardia* more tolerant to reactive oxygen species which are generated by redox cycling of furazolidone nitroradical anions. Still, it is questionable whether these enhanced enzyme activities can account for the far more pronounced resistance induced in vitro [61]. Importantly, strains with previously induced furazolidone resistance can be adapted to high concentrations of quinacrine much easier than wild type, suggesting the existence of a common drug export mechanism [45]. However, *Giardia* laboratory cell lines with metronidazole resistance also tolerate more furazolidone [74], albeit to a far lesser degree as compared to metronidazole.

### Resistance to Quinacrine

Only few data on quinacrine resistance in *G. lamblia* are available. However, in a careful study resistance to quinacrine was gradually induced in vitro in several strains [45]. Eventually, resistant cell lines were able to grow at concentrations about 50-fold higher than the normal IC_50_ [88]. Fluorescence analysis suggested that the drug is excluded from resistant *G. lamblia* [45].

## Conclusions

It is unlikely that the management of giardiasis will be compromised in the future by a lack of treatment options. Although treatment failures and drug resistance in *Giardia* can be problematic, a comparably large number of effective drugs are available, and combination therapy is almost always successful. Moreover, promising novel treatment options have been presented recently, such as formononetin [89, 90], auranofin [5•] and fumagillin [6•], and natural products with antigiardial activity have remained an as yet untapped source [91].
